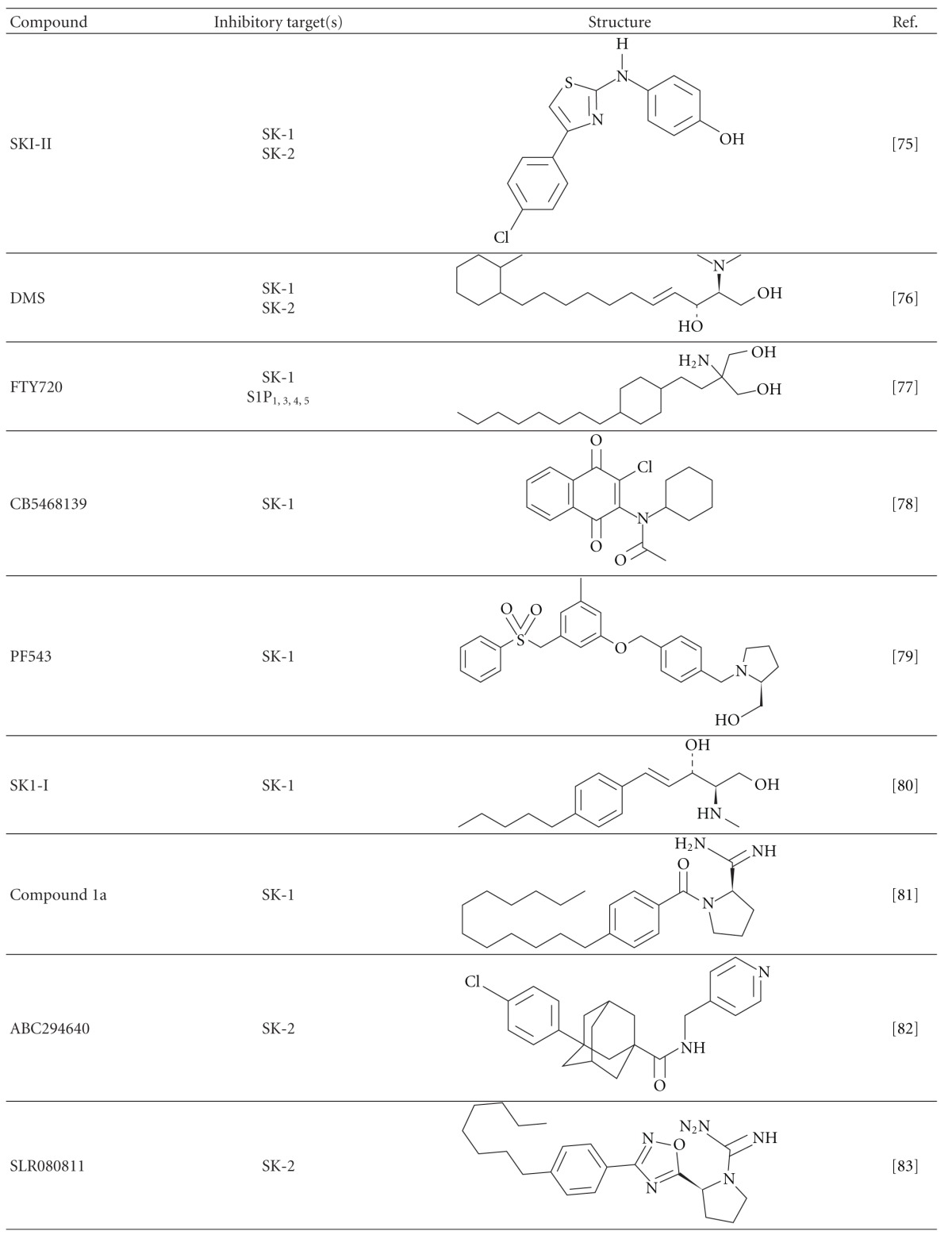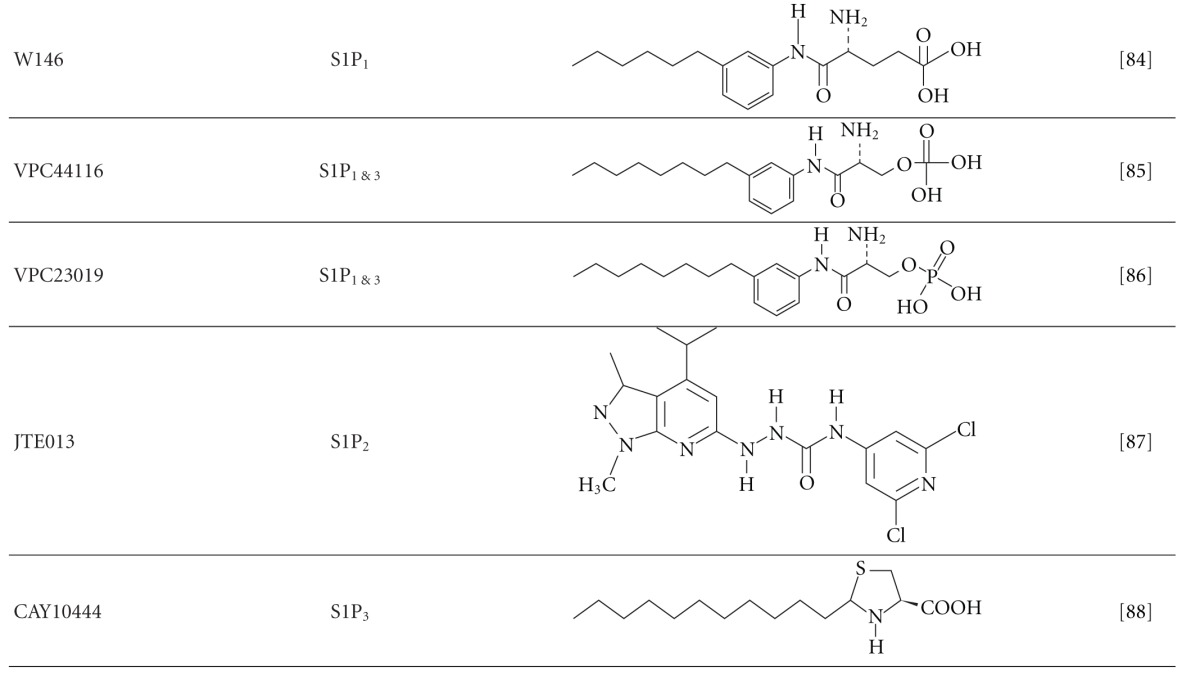# Sphingolipids: A Potential Molecular Approach to Treat Allergic Inflammation

**DOI:** 10.1155/2012/154174

**Published:** 2012-12-18

**Authors:** Wai Y. Sun, Claudine S. Bonder

**Affiliations:** ^1^Centre for Cancer Biology, SA Pathology, Frome Road, Adelaide, SA 5000, Australia; ^2^School of Medicine, University of Adelaide, Adelaide, SA 5000, Australia; ^3^Cooperative Research Centre for Biomarker Translation, La Trobe University, Bundoora, VIC 3086, Australia; ^4^School of Molecular and Biomedical Sciences, University of Adelaide, Adelaide, SA 5000, Australia

## Abstract

Allergic inflammation is an immune response to foreign antigens, which begins within minutes of exposure to the allergen followed by a late phase leading to chronic inflammation. Prolonged allergic inflammation manifests in diseases such as urticaria and rhino-conjunctivitis, as well as chronic asthma and life-threatening anaphylaxis. The prevalence of allergic diseases is profound with 25% of the worldwide population affected and a rising trend across all ages, gender, and racial groups. The identification and avoidance of allergens can manage this disease, but this is not always possible with triggers being common foods, prevalent air-borne particles and only extremely low levels of allergen exposure required for sensitization. Patients who are sensitive to multiple allergens require prophylactic and symptomatic treatments. Current treatments are often suboptimal and associated with adverse effects, such as the interruption of cognition, sleep cycles, and endocrine homeostasis, all of which affect quality of life and are a financial burden to society. Clearly, a better therapeutic approach for allergic diseases is required. Herein, we review the current knowledge of allergic inflammation and discuss the role of sphingolipids as potential targets to regulate inflammatory development *in vivo* and in humans. We also discuss the benefits and risks of using sphingolipid inhibitors.

## 1. Introduction

Allergic inflammation can occur rapidly or delayed via the classical inflammatory immune reaction involving the production of specific IgE antibodies as well as the activation of inflammatory cells and the endothelium [1]. Many proinflammatory mediators and cytokines including histamine, leukotriene, and tumor necrosis factor *α* (TNF*α*) can activate the vascular endothelial cells (ECs) to cause pro-inflammatory microvasodilation and mediate leukocyte recruitment from the circulation to the sites of allergic inflammation [2, 3]. Excessive and prolonged leukocyte recruitment can result in extracellular matrix (ECM) remodelling and tissue damage [4]; thus controlling EC activation provides a strategy to minimize allergic inflammation. This review discusses the pathophysiology of vascular ECs during allergic inflammation, current treatments and new therapeutic approaches. We focus on the role of sphingolipids in the regulation of vasculature during the early phase of allergic inflammation, in particular, studies utilizing sphingolipid knockout animals which support their potential as new therapeutic targets.

## 2. Pathophysiology in Acute Allergic Inflammation

**Figure 1:**
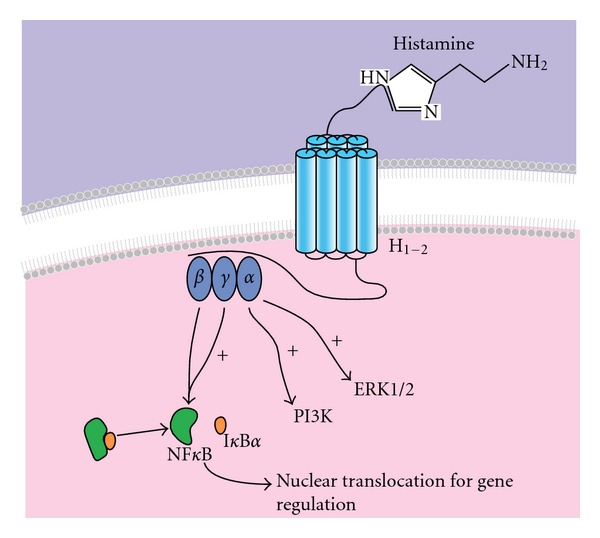
Histamine receptors on ECs. Two histamine receptors (H_1_ and H_2_) are found on ECs. Within minutes of histamine binding to its receptors, the G-protein subunits are activated to initiate intracellular signalling. The *α*q subunit of the G-protein contributes to reduced cAMP accumulation, induced ERK1/2, and induced inositol phospholipid (PI3K) signalling. The *β* and *γ* subunits contribute to the activation of NF*κ*B and subsequent translocation into the nucleus where transcriptional processes are regulated causing cellular changes, such as vascular contraction and permeability, all of which are important for immune regulation and inflammation.

Histamine is a potent proinflammatory mediator primarily released by mast cells and basophils with up to 0.01–1 mol/m^3^ found in the periphery during an allergic response [5, 6]. Histamine mediates dendritic cell maturation [7], T lymphocyte differentiation and migration [8–10], and endothelial cell proliferation [11] via a family of four G-protein-coupled receptors (H_1-4_) [12]. Histamine receptors are differentially expressed with only H_1_ and H_2_ expressed by vascular ECs [13, 14] (Figure 1). Within minutes of histamine exposure and binding to H_1_ and H_2_, the G-protein subunit *α*q is recruited to decrease cAMP accumulation and subsequent EC contraction [15]. By contrast, the G protein *β* and *γ* subunits are activated to induce the nuclear factor kappa-light-chain-enhancer of activated B cells (NF*κ*B) [16]. Ligand interaction with the H_1_ receptor causes vascular permeability, synthesis of prostacyclin and platelet activating factor, and release of von Willebrand Factor (vWF) and nitric oxide [17, 18]. H_2_ receptor stimulation is linked to the G*α*s subunit for the activation of adenylate cyclase and formation of cyclic adenosine monophosphate (cAMP), which induces intracellular calcium-mediated vasodilatation at a slower rate of onset than that of H_1_ receptor [19, 20]. In addition, the H_2_ receptor can negatively regulate the release of histamine by mast cells and basophils [21] and suppress the production of TNF*α* and IL-12 from inflammatory cells [10, 22, 23]. 

## 3. Antihistamines as the Current Mainstay Treatment for Allergic Inflammation

Antihistamines (e.g., diphenhydramine and chlorpheniramine) were first developed in the 1930s as an inverse agonist for the histamine receptors and have been commonly used to treat and prevent allergic symptoms ever since [24] (Table 1). Patients treated with H_1_ antihistamines exhibit reduced production of histamine and leukotrienes as well as downregulation of adhesion molecule expression on the vasculature which in turn attenuates allergic symptoms by 40–50% [20, 25–28]. Long term treatment with H_1_ antihistamines can retard the progression of respiratory disease by inactivating functions of macrophages and other Th2 cells thus preventing local tissue remodelling and damage [29, 30]. Second- and third-generation antihistamines (e.g., loratadine, fexofenadine, and cetirizine) (Table 1) were generated in the 1980s. These drugs also target the H_1_ receptor but, in general, are less lipophilic and therefore exhibit reduced ability to penetrate the blood-brain barrier resulting in a less sedating effect than the first generation counterparts [28, 31]. Notably, 2–5 times higher dose of these second-generation antihistamines are required to control mild seasonal allergic symptoms when compared to the first-generation medications [32]. Using H_1_ antihistamines at a high dose remains controversial as (i) animal studies have shown that mice treated with high doses of fexofenadine during the allergen challenge exhibited reduced lung inflammation, reduced Th2 responses, and reduced the secretion of IL-4, -5, and -13 [7, 29], (ii) a recent human clinical study demonstrated that high-doses of desloratadine only marginally improved allergic symptoms in patients without an increase in adverse effects when compared to the standard doses [33] and (iii) long-term high-dose use of antihistamines in patients with chronic urticaria retained adverse effects, such as rapid eye movement, sleep disturbance, and negative impacted on learning and performance [34]. Clearly, other effective clinical approaches are needed to combat allergic inflammation.

## 4. Antiselectin Therapy for Inflammatory Diseases

Another approach is to target the expression of adhesion molecules on ECs, such as selectins, which are known to initiate the early capturing and rolling of leukocytes from the circulation. Antagonism of the selectins is recognized to be a therapeutic approach to prevent and minimize inflammatory reactions. Evidence for this comes from P-selectin-deficient mice which, when challenged with the inflammatory irritant thioglycollate, exhibit attenuated leukocyte rolling in the blood vessels for up to 4 hours [35]. They also exhibit a significant reduction in leukocyte infiltration at the inflammatory hindlimb by ischemia on postoperative day 14 when compared to wildtype (WT) controls [36]. In humans, the recruitment of activated neutrophils to the local inflamed tissue is largely dependent on adhesion molecules as evidenced by patients with leukocyte adhesion deficiency (LAD II) whose neutrophils lack functional sialyl Lewis X expression (a fucose-containing glycoconjugate ligand for P-, E-, and L-selectin), exhibit reduced rolling and firm adhesion on the endothelium [37]. Together, these show that controlling expression of adhesion molecules can influence the early phase as well as the chronic phase of inflammatory reactions. 

 Selectin antagonists have been examined in preclinical studies, including cutaneous inflammation, allergy and ischemia-reperfusion injury [38, 39]. The first selectin antagonist CY1503 (Cylexin), an analogue of sialyl Lewis X which inhibits E-, P-, and L-selectins, has demonstrated a reduction in the degree of myocardial infarct size associated with a canine model of coronary artery ischemia and reperfusion, and reduced leukocyte accumulation at 4.5 hours after operation [40]. However, the effects of CY1503 remain controversial as a second similar study failed to consistently reduce myocardial injury and neutrophil accumulation at 48 hours post-operation [41]. Treatment with CY1503 also failed to attenuate the “no-reflow” phenomenon of leukocytes and could not limit the myocardial infarct size in the rabbit [42]. More recently, the oral P-selectin blocking agent, Pentosan Polysulfate Sodium (PPS), has been examined in a Phase I clinical study, wherein a single dose of PPS showed improvement of microvascular blood flow in patients with sickle cell disease [43]. However, no study to date has examined the efficacy of PPS in controlling leukocyte recruitment during allergic inflammation.

To date, four classes of selectin blocking agents have been developed: (i) carbohydrate based inhibitors targeting all P-, E-, and L-selectins [44], (ii) antihuman selectin antibodies [45], (iii) a recombinant truncated form of PSGL-1 immunoglobulin fusion protein [46], and (iv) small-molecule inhibitors of selectins [47]. Notably, most of the selectin blocking agents have failed in phase II/III clinical trials or the clinical studies were ceased due to their unfavorable pharmacokinetic properties and high cost [39]. Animal models also suggest that the timing and potency of selectin blockade are crucial to preventing the development of allergic inflammation with a greater than 90% reduction in leukocyte rolling required for firm adhesion events to be significantly attenuated [48, 49]. Given that the direct selectin blockade by the current compounds remains unsuccessful to regulate allergic inflammation, new therapeutic approaches which target the regulation and expression of adhesion molecules are warranted. 

## 5. Sphingomyelin Pathway

**Figure 2:**
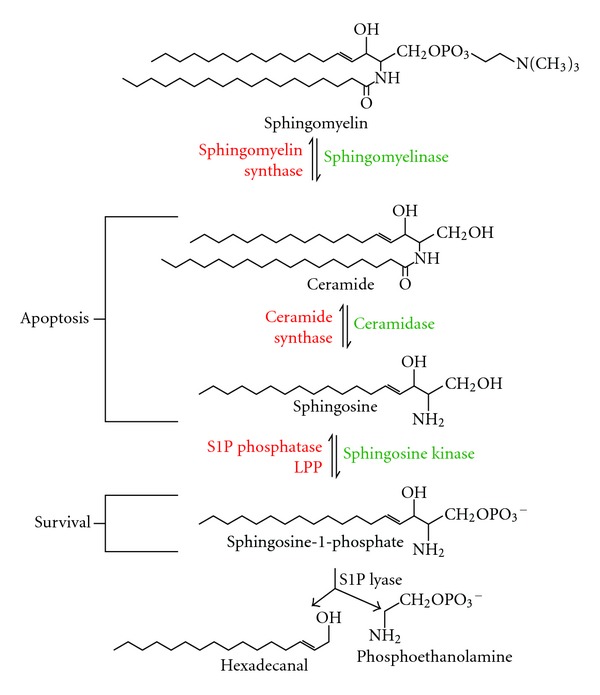
Sphingomyelin pathway. Sphingomylein is hydrolysed to ceramide, which is then metabolized to sphingosine and sphingosine-1 phosphate (S1P) by different kinases (green). This process is reversible via the activities of different synthases and phosphatases (red). The levels of the biological product, S1P, are regulated by S1P lyase which degrades it into hexadecanal and phosphoethanolamine. Although the structures of each sphingolipid are similar, they have divergent cellular functions with ceramide and sphingoine being pro-apoptotic, and S1P being prosurvival.

The lipid enzyme, sphingosine kinase (SK), was originally identified for its role in the sphingomyelin degradation pathway but is increasingly being recognized as an important signalling molecule (Figure 2). There are excellent reviews focusing on the roles of SK/S1P in diseases, such as cancer [50], immunity [51], asthma [52], multiple sclerosis [53], rheumatoid arthritis [54], and pancreatic islet transplantation [55]. Herein, we discuss how SK can be used as a new therapeutic target to combat allergic inflammation, referencing animal models and human trials, together with the benefits and adverse effects of manipulating SK using inhibitors. 

## 6. Sphingosine Kinase

Two isoforms of SK (i.e., SK-1 and SK-2) have been cloned and characterized in mammalian cells, which both catalyze the phosphorylation of sphingosine to form sphingosine-1-phosphate (S1P) [56, 57]. SK-1 has been shown to be the primary contributor to serum S1P levels with *SphK1−/−* mice exhibiting a ~50% reduction in serum S1P when compared to wildtype (WT) mice [58] and the *SphK2−/−* mice serum S1P levels exhibiting no reduction. In fact, Zemann et al. showed an increase in serum S1P of *SphK2−/−* mice [59]. Notably, S1P was undetectable in plasma and lymph of the conditional double knockout mice [60].

**Figure 3:**
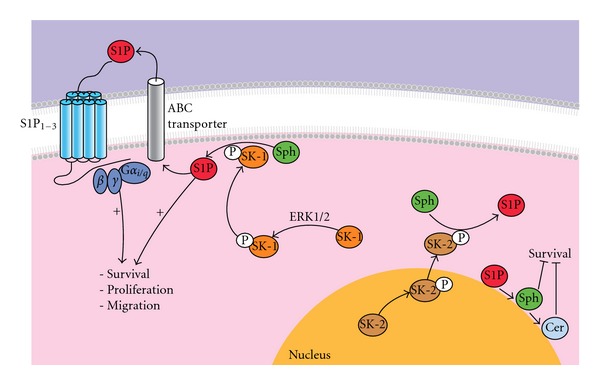
Intracellular SK-1 and SK-2 activity. The activation of SK-1 and SK-2 occurs via ERK1/2 phosphorylation in response to proinflammatory mediators, such as histamine and TNF*α*. Upon the activation, SK-1 is translocated from the cytoplasm to plasma membrane where it catalyses sphingosine to form S1P. S1P can then be transported outside the cell and then act back on its receptors to induce the activation of G-proteins for subsequent cellular changes, such as survival, proliferation, and migration. In contrast, SK-2 activity is associated primarily with the nuclear membrane, where it is phosphorylated prior to being translocated out of the nucleus. At the nuclear membrane and endoplasmic reticulum, S1P can be dephosphorylated to sphingosine and ceramide via the sphingolipid salvage pathway where many enzymes including sphingomyelinases, cerebrosidases, ceramides, and ceramide synthases are involved to induce apoptosis.

The polypeptide sequences of SK-1 and SK-2 contain 80% similarity, which supports compensatory effects when one isoform of SK is knocked down [56, 57]. Interestingly, the localization of SK-1 and SK-2 differs with SK-1 being predominantly found in the cytoplasm and at the plasma membrane leading to prosurvival effects [61, 62], and SK-2 being predominantly found in the nucleus and at the endoplasmic reticulum (ER) promoting proapoptotic effects [63, 64] (Figure 3). Three splice isoforms of SK-1 have been identified (i.e., SK-1a, SK-1b, and SK-1c) that differ at their N-termini with additional 14 and 86 amino acids in SK-1b and SK-1c, respectively [65]. Two variants of SK-2 have also been identified (i.e., SK-2 and SK-2 long (SK2L)) arising from alternate start sites [57]. The specific physiological role for each SK variant is yet to be further elucidated. 

 SK has intrinsic activity and can be further activated by many biological stimuli, including histamine [66], cross-linking of immunoglobulin receptors [11], TNF*α* [67], vascular endothelial growth factor (VEGF), interleukins, complement C5a [68], and bradykinin [11]. Upon stimulation, the catalytic activity of SK-1 increases via the phosphorylation of extracellular signal regulated kinase (ERK)-1/2 at Ser225 which results in the translocation to the inner plasma membrane [69]. The binding of SK-1 to lipid phosphatidylserine can enhance SK-1 activity and plasma membrane translocation [70]. More recently, calcium- and integrin-binding protein (CIB)-1 protein has been identified to translocate SK-1 to the plasma membrane [71]. Conversely, dephosphorylation at Ser225 causes deactivation of basal and TNF*α*-induced SK-1, a process shown to be regulated by protein phosphatase 2A (PP2A) [72, 73]. In contrast, SK-2 does not possess the Ser225 phosphorylation site but its activation, also via the ERK pathway, is suggested to occur by phosphorylation at Ser351 and Thr578, which induces translocation from the nucleus to endoplasmic reticulum [57, 74].

## 7. Sphingosine-1-Phosphate

S1P is the biological product of SKs and is predominantly formed in the cytoplasm. S1P can be retained intracellularly or released by platelets, neutrophils, leukocytes, ECs, and mast cells via the transporters, ATP-binding cassette (ABC) transporter ABCC1, ABCA1 and ABCG1 [89–92]. S1P is bound to high-density lipoproteins (HDL) and plasma proteins, such as albumin, which stabilizes S1P in the circulation [93]. Platelets secrete the highest levels of S1P but ECs also upregulate their release of S1P in response to activation and shear stress [94]. The concentration of S1P ranges from 4 × 10^−4^ to 1.2 × 10^−3^ mol/m^3^ in serum, 2 × 10^−4^ to 5 × 10^−4^  mol/m^3^ in plasma, and 5 × 10^−7^ to 7.5 × 10^−6^ mol/m^3^ in tissue [93, 95–97]. Interestingly, S1P can also be formed outside the cell as SK-1 has been shown to be secreted by human umbilical vein ECs (HUVEC) and macrophages [98, 99]. 

Increasing evidence supports intracellular targets for S1P signalling with S1P binding to histone deacetylases (HDAC)-1 and -2 to regulate histone acetylation [100], TNF receptor-associated factor 2 (TRAF2) to regulate inflammation, antiapoptotic and immune responses via the NF*κ*B pathway [101], and prohibitin 2 (PHB2) for regulation of mitochondrial assembly and function [102]. By contrast, extracellular S1P-mediated signalling has been well described with five S1P receptors (S1P_1, 2, 3, 4, 5_) coupled with various G*α* proteins (e.g., G*α*_i_, G*α*_q_, and G*α*_12/13_) which activate different downstream targets, such as PI3 K/Akt, Bcl2, adenylyl cyclase, ERK, phospholipase C, and p53 for cellular responses in both an autocrine and paracrine manner [103–107]. Briefly, S1P_1_ is important to regulate the egress of lymphocytes into the blood stream [108], and S1P_2_ is involved in mast cell degranulation and recovery from anaphylaxis *in vivo* [109, 110], S1P_3_ is involved in vascular development in the embryo [111]. S1P_4_ and S1P_5_ are not well studied but have been shown to be expressed by dendritic cells and lymphocytes, respectively [112, 113].

## 8. Genetic Manipulation of SK/S1P *In Vivo*

To investigate the physiological roles of SK/S1P *in vivo* and whether their manipulation can regulate disease development, genetically modified mice with depletion of either SK-1 or SK-2 gene (*Sphk1* or *Sphk2*) have been generated and no phenotypical abnormalities have been identified under normal conditions [58, 77]. By contrast, the depletion of both *Sphk1* and *Sphk2* is embryonic lethal by day 13.5 due to the severe defects in vasculogenesis and neurogenesis involved in CNS development [114]. More recently, the *Sphk1* and *Sphk2* heterozygous-knockout mice (i.e., *Sphk1*−/−*Sphk2*+/−) have been generated [115]. Although *Sphk1−*/*−Sphk2*+/− mice have not been studied extensively, the female mice exhibit a significant breakage of blood vessels in the uterine causing early pregnancy loss, which suggests that a basal level of SK is required for blood vessel integrity or stability [115]. To investigate the inhibitory effects of both SKs, administration of specific SK inhibitors serves as an alternative approach to attain the double knockdown effects, for example, administration of ABC294640 (SK-2 specific inhibitor) to *SphK1−/−* mice and administration of CB5468139 (SK-1 specific inhibitor) to *SphK2−/−* mice. However, studies using this alternative approach are lacking, which are likely due to the complicated pharmacokinetics and pharmacodynamic of the SK inhibitory agents *in vivo*. 

## 9. SK/S1P in Allergic Inflammation

SK and S1P are involved in multiple cellular functions, such as survival, differentiation, activation and migration (reviewed in [107]). Notably, these cellular properties are involved in many disease developments, including allergic inflammation. To better understand the role of SK/S1P in allergic inflammation, a number of studies have examined the specific roles of each SK isoform and S1P receptors via genetically modified mice. For example, both *Sphk1−/−* and *Sphk2−/−* mice have been shown to exhibit a reduction in ovalbumin (OVA)-induced IgE and IgG production via an inability to increase mast cell protease 1 in response to OVA, an enzyme required for IgE-induced anaphylaxis [116]. Our recent work has shown that *Sphk1−/−* mice but not *Sphk2−/−* mice exhibit an attenuated histamine-induced P-selectin expression and neutrophil recruitment [66]. This is in agreement with a study by Baker et al. who generated hTNF/*Sphk1−/−* mice (i.e., *Sphk1−/−* mice carrying the human modified copy of TNF*α*) and showed that only hTNF/*Sphk1−/−* mice but not hTNF/*Sphk1+/+*, hTNF/*Sphk1−/+,* or hTNF/*Sphk2−/− *mice exhibited a reduction in paw inflammation and bone deformity [117, 118]. Moreover, this was determined to be due to decreased articular COX2 protein and Th17 cell contribution to inflammation [117]. In terms of recovery from allergic inflammation, *Sphk1−/−* and S1P_2_ *−*/*−* mice were observed to have increased vasodilation, poor recovery from anaphylaxis and delayed clearance of histamine. This was not observed in the *Sphk2−/−* mice [109]. Administration of S1P to *Sphk1−/−* mice can rescue these phenomena, which suggests that SK-1 activity aids in the recovery from anaphylaxis [109].

In humans, increasing evidence suggests that SK and S1P are involved in the pathophysiology of inflammatory diseases, such as asthma [119], chronic obstructive pulmonary disease (COPD) [120], microbial-induced sepsis [121], acute pancreatitis [122], and rheumatoid arthritis [123]. Studies have shown that the SK-1 protein and activity are upregulated markedly in peripheral immune cells including neutrophils, lymphocytes, and macrophages during the early phase of these diseases, which allow for their activation and release of the proinflammatory cytokines TNF*α*, IL-1*β* and IL-6 [121, 122]. Not surprisingly, high levels of S1P were detected in the synovial fluid of arthritic patients, which enhances COX-2 expression and prostaglandin E(2) production via the S1P_1_ receptor [123]. Blockade of SK-1 in tissue samples extracted from these patients exhibited a decrease in proinflammatory cytokine expression [121], which suggests that the regulation of SK-1/S1P pathway is a potential therapy for inflammatory diseases.

## 10. Pharmacological Manipulation of SK/S1P 

There are a number of SK and S1P receptor inhibitors that have been generated and studied in the last few decades (Table 2) (reviewed in [124, 125]). Blockade of SK-1 by inhibitors can attenuate prostate cancer [65], melanoma [126], inflammation in rheumatoid arthritis [123] and asthma [127] *in vivo*. Of all of the SK/S1P inhibitors, only a few have proceeded to clinical trials and been approved for human use based on their pharmacokinetics, target specificity, efficacy, adverse effects, and safety profile. The best example to date is FTY720 (Fingolimod), which was the first oral prodrug to be approved by the Food and Drug Administration (FDA) and Therapeutic Goods Administration(TGA) for the clinical treatment of multiple sclerosis (MS) [128]. The first described mechanism of FTY720 is predominantly phosphorylated by SK-2 to form FTY720-P, which is then able to bind to S1P receptors (S1P_1, 3, 4, 5_) [77, 129]. In MS, FTY720-P blocks S1P signalling largely by the internalization of the S1P_1_ on lymphocytes causing lymphocyte egress from the lymphoid organs and lymphopenia in the periphery [108]. 

Interestingly, later studies have shown that FTY720 without phosphorylation can potently inhibit SK-1 by competing with sphingosine as a substrate for SKs and thereby preventing subsequent S1P formation [129–131]. Furthermore, the analogues of FTY720 (i.e., (S)- and (R)- FTY720-vinylphosphonate) bind to an allosteric site of SK-1 to induce proteasomal degradation in cells in a noncompetitive manner [132]. As FTY720 itself can inhibit SK-1, studies have also examined whether high concentrations (larger than the recent clinical dose of 0.5 mg once daily) and multiple dosing of FTY720 can be a potential therapy for cancer and renal transplantation [133, 134]. Unfortunately, results showed that FTY720 does not improve the prognosis for postrenal transplantation when compared to the current protocols [134, 135], likely due to the multiple inhibitory effects of FTY720 on S1P receptors, SK-1, autotoxin, protein phosphatase 2A, ceramide synthases, S1P lysase, protein kinase C and cytosolic phospholipase A [reviewed in [136]]. Clearly, new and specific SK/S1P inhibitors are required. To this end, Schnute et al. recently generated a specific and potent SK-1 inhibitor, PF-543, which inhibits SK-1 by competing with sphingosine and resulting in rapid reduction of S1P formation [79]. The inhibitory effect of SK-1 by PF-543 is over 1000-fold more potent than other SK inhibitors such as N,N-dimethylsphingosine (DMS) and SKI-II. However, the efficacy of PF-543 *in vivo* remains to be examined. In addition, Kharel et al. reported that their two new amidine-based SK-1 inhibitors (1a and 1b) can selectively inhibit SK-1 at high potency for rapid reduction in S1P levels without toxicity *in vitro* and *in vivo* [81].

Although SK-2 is less well studied than SK-1, a role for SK-2 (via the administration of the SK-2 inhibitor, ABC294640) has been described in tumor development [82, 137], Crohn's disease [138], hepatic ischemia-perfusion [139], and osteoarthritis [140]. However, this SK-2 inhibitor also binds to oestrogen receptor [141], which suggests that administration of this compound may result in additional off-target effects. Interestingly, a new selective SK-2 inhibitor, SLR080811, has been shown to inhibit SK-2 at a higher potency than ABC294640 *in vitro* and drive an SK-1-dependent increase in blood S1P in WT mice [83]. Whether this small molecule is suitable for the clinic still requires long-term efficacy and safety data development. 

Notably, pharmacological manipulation of SK/S1P does not always lead to the same results as observed for genetic manipulation *in vivo*. As mentioned above, the hTNF/*Sphk2−/−* mice exhibited no significant difference in arthritic inflammation when compared to controls [118]. However, the hTNF mice treated with ABC294640 exhibited severe arthritic inflammation in the same study, which may suggest that high dose of the agent and acute inhibition of SK-2 contribute to this phenomenon [118]. Moreover, other animal models include that thioglycollate-induced peritonitis and collagen-induced arthritis (CIA) have shown that the recruitment of neutrophils and lymphocytes to sites of inflammation in *Sphk1−/−* mice did not differ from that of WT mice [142]. By contrast, Lai et al. have shown that knockdown of either SK-1 protein or gene in mice by DMS and small interfering (si)RNA, respectively, exhibit reduced CIA severity [123, 143]. These different observations may be due to the different time period of stimulus challenge, animal strains and models for susceptibility. Nevertheless, taken together these studies clearly indicate that SK and S1P are involved in the development of allergic inflammation. 

## 11. Adverse Effects of SK Inhibition

The inhibition of SK/S1P pathway may be an effective therapeutic approach to control allergic diseases as shown by the *in vivo* studies discussed above. However, excessive or prolonged blockade of SK/S1P may lead to profound adverse effects as evidenced by S1P_1_ *−/−* and double knockout of *Sphk1−/− Sphk2−/−* animals being embryonic lethal [106, 114] as well as S1P_2_ *−/−* mice being deaf [144] and experiencing occasional seizures [145]. The “side effects” of small molecule therapy that modulate the SK/S1P pathway may also raise concerns. For example, FTY720 at the clinical dose has been reported to cause transient bradycardia, atrioventricular block, macula oedema, hypertension, dyspnea, and elevated liver enzymes [146]. These symptoms are infrequent and manageable; however, compliance of this treatment can be discouraged by patients. In addition, treatment with FTY720 is also thought to increase the risk of infections as *Sphk1−/−* mice are more susceptible for endotoxin-induced lung inflammation than WT controls [147]. However, human preclinical data showed that FTY720-treated patients have no increased risk of infections in 2-year treatment when compared to the placebo group, except a small increased risk of lower respiratory tract and lung infections [128]. Notably, although the regulation of SK/S1P looks promising for controlling disease development, high specificity and potency of the pharmacological agents are preferable to avoid the undesirable off-target effects. 

## 12. Strategy for Targeting Sphingolipids as a Therapeutic Approach

**Figure 4:**
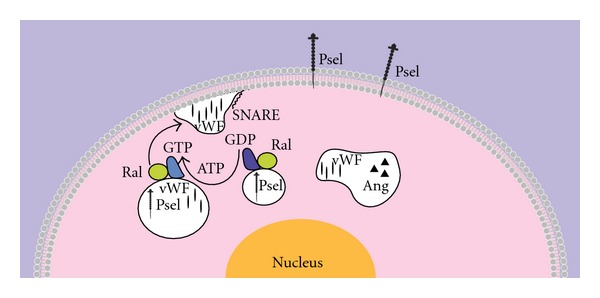
. Exocytosis of P-selectin by ECs. P-selectin is preformed and stored in Weibel Palade bodies (WPBs). It is found to be solely present or co-stored in WPBs with von Willebrand Factor (vWF) or angiopoietins (Ang). Upon extracellular stimulation, WPBs exocytose to the cell surface via the activation of Ral-GTP from Ral-GDP. WPB-containing vWF is also driven and translocated to the plasma membrane by SNARE. The rapid surface expression of P-selectin mediates the initial recruitment of leukocytes to ECs by rolling and tethering, which is important during the early development of allergic inflammation.

An effective approach to target sphingolipids for allergic inflammation diseases and avoid adverse effects is to better understand “when” and “where” such that specific SK/S1P inhibitors can be administrated appropriately. In ECs, we and others have demonstrated that the SK/S1P pathway regulates the expression of adhesion molecules to control neutrophil recruitment* in vitro *and *in vivo* (Figure 4). For example, during the early phase of allergic inflammation, histamine-induced SK-1 activity (but not SK-2 activity) rapidly exocytoses P-selectin to the surface of ECs to initiate neutrophil rolling in the postcapillary venules of WT mice, a process shown to be S1P receptor independent [66]. As expected, this histamine-induced neutrophil recruitment does not occur in *Sphk1−/− *mice [66]. Furthermore, TNF*α*-induced SK-1 activates *α*_5_ *β*_1_ integrin on human umbilical vein ECs (HUVEC) to promote the adhesion of neutrophils under shear stress, again the events appear to be S1P receptor independent and can be inhibited by FTY720 [148]. 

By contrast in the late phase of allergic inflammation (>4 hours), S1P receptor-activated pathways promote vascular adhesion molecule (VCAM)-1, intercellular adhesion molecule (ICAM)-1, and E-selectin gene and protein expression on HUVEC in response to TNF*α* [67], globular adiponectin [149], or histamine [150]. Exposure of ECs to S1P can also increase Weibel Palade body (WPB) exocytosis of vWF in a PLC-*γ*-induced calcium-dependent manner. However, prolonged exposure of S1P enhances PI3K-induced nitric oxide production resulting in reduced WPB exocytosis by ECs [151]. Taken together, these studies suggest that increased SK-1 activity is predominantly involved in the early phase of allergic inflammation whilst S1P/S1P receptors are primarily involved in more delayed immune responses.

S1P_1–5_ are distributed in different tissues with S1P_1–3_ being widely expressed and at high levels in brain, lung, spleen, heart, liver, skeletal muscle, and kidney with addition of S1P_1_ in lymphoid and S1P_3_ in testis; S1P_4_ is restricted to lymphoid and lung tissue and S1P_5_ is only expressed in brain, skin, and spleen (reviewed in [152]). These divergent tissue distributions of S1P receptors may provide some insight into which specific S1P receptor inhibitors should be administered in relation to the development of inflammation and disease. Notablty, FTY720-P binds to S1P_1, 3, 4, 5_ and may result in multiple side effects; thus other selective S1P_1_ inhibitors (ONO-4641 and CS-0777) have been generated and undergone Phase 1 and 2 clinical trials for MS and psoriasis (reviewed in [125, 153]). Different methods of administration can be used to deliver the inhibitors/drugs for local inhibitory effects as evident by *in vivo* studies where the inhalation of SK inhibitor can attenuate airway inflammation [127], the administration of FTY720 in the eyes can prolong corneal graft survival [154], and nanoparticle-mediated delivery of drugs can enhance the therapeutic outcomes in hindlimb ischemic mice [155]. However, many questions remain to be answered, such as whether this nano-technology is effective enough to deliver SK/S1P inhibitors to specific sites of the body and whether it is safe to be used in humans.

## 13. Conclusion and Future Perspectives

 Early allergic reactions and recruitment of inflammatory cells are key to allergic disease formation and progression. An effectual therapeutic approach is lacking amongst the current treatment options, and most treatments (e.g., H_1_ antagonists) are ineffective in their regulation of the early phase of allergic inflammation. Thus a better therapeutic strategy is urged for a rapid control of allergic symptoms to prevent tissue damage and development of severe conditions. The SK/S1P pathway has been shown to be important in cell survival, migration, differentiation, and immune responses. Herein, we discuss its role in allergic inflammation, both the early and late phases as well as chronic inflammation. Further studies involving the manipulation of SK/S1P pathway and its impact on a variety of diseases as well as the early phase of allergic inflammation will culminate to provide better insight into how we can translate animal studies into a new clinical treatment for human allergic inflammation.

Based on these *in vitro* and *in vivo* studies, sphingolipids are clearly involved in the regulation of adhesion molecule expression on the vasculature and as such may be a biological marker for attenuating leukocyte recruitment and subsequent allergic inflammatory reactions. The next step is to translate these animal models into human clinical studies with the ultimate goal of developing new treatments to tackle allergic diseases. Herein we propose that the current sphingolipid compounds may be effective in attenuation of allergic inflammation. For example, FTY720 or new small molecular inhibitors could be further investigated for their drug adverse effect profile to then determine their suitability for long-term use as prophylaxes.

## Figures and Tables

**Table 1:** Common antihistamines marketed in Australia.

Some common antihistamines			
First generation	Second generation		Third generation
Systemic	Systemic	Topical	Systemic/topical
Promethazine	Cetirizine	Azelastine	Levocetirizine
Pheniramine	Loratadine	Levocabastine	Desloratadine
Cyproheptadine	Terfenadine		Fexofenadine
Dexchlorpheniramine	Ketotifen		
Trimeprazine	Mizolastine		

**Table 2:** Synthetic inhibitors of SK and S1P receptors.